# Establishment of a 3D Multicellular HCC Tumor Spheroid Model to Unravel Nrf2’s Influence on the Tumor Immune Microenvironment

**DOI:** 10.3390/bioengineering13030336

**Published:** 2026-03-13

**Authors:** Nicole Böttcher, Philipp Krumm, Rosanna Huchzermeier, Lara Berschkeit, Johanna Vollmer, Julie Dick, Thomas Pufe, Athanassios Fragoulis

**Affiliations:** 1 Department of Anatomy and Cell Biology, Uniklinik RWTH Aachen, 52074 Aachen, Germany; nischroeder@ukaachen.de (N.B.); pkrumm@ukaachen.de (P.K.); rhuchzermeie@ukaachen.de (R.H.); lara.bednarek@alumni.fh-aachen.de (L.B.); jvollmer@ukaachen.de (J.V.); julie.dick@rwth-aachen.de (J.D.); tpufe@ukaachen.de (T.P.); 2 Department of Internal Medicine I, University Hospital Aachen, RWTH Aachen University, 52074 Aachen, Germany; 3 Institute for Molecular Cardiovascular Research (IMCAR), Uniklinik RWTH Aachen, RWTH Aachen University, 52074 Aachen, Germany; 4 Aachen-Maastricht Institute for Cardio-Renal Disease (AMICARE), Uniklinik RWTH Aachen, RWTH Aachen University, 52074 Aachen, Germany

**Keywords:** Nrf2, spheroid, multicellular tumor spheroid, HCC, N-HCC25, tumor associated macrophages, tumor immune microenvironment, 3D cell culture

## Abstract

Hepatocellular carcinoma (HCC) remains a leading cause of cancer-related death, yet adequate *in vitro* models mimicking the tumor immune microenvironment (TIME) are rare. Specifically, the role of the transcription factor nuclear factor erythroid 2-related factor 2 (Nrf2) in modulating interactions between tumor cells and tumor-associated macrophages (TAMs) is not fully understood. We established a 3D multicellular tumor spheroid (MCT) model using murine N-HCC25 cells with CRISPR/Cas9-mediated knockouts of Nrf2 and its negative regulator Kelch-like ECH-associated protein 1 (Keap1), the latter mimicking constitutive activation. N-HCC25 cells were co-cultured with bone marrow-derived macrophages (BMDMs) isolated from wild-type and Nrf2-knockout C57BL/6J mice. We compared co-culture setups (conditioned media, transwell systems, direct contact) using RT-qPCR, flow cytometry, and invasion assays. 3D spheroid systems better preserved stemness than 2D cultures and revealed functional Nrf2-dependent effects such as increased Vegf-α secretion in Keap1-deficient spheroids. Among the different co-cultivation models, the most profound effects were observed in the MCT model. Macrophages successfully integrated into the spheroids and triggered invasive outgrowth, whereas MCTs containing Nrf2-deficient macrophages displayed markedly reduced tumor spheroid growth and lower programmed cell death ligand-1 expression. These findings demonstrate that Nrf2 signaling in macrophages fosters an immunosuppressive and pro-invasive microenvironment. The established MCT model provides a suitable platform to further unravel Nrf2-dependent mechanisms in the HCC TIME.

## 1. Introduction

With more than 750,000 deaths worldwide each year, liver cancers, in general, represent one of the third deadliest malignant cancer types [1,2,3]. A significant contributor to these cases is the hepatocellular carcinoma (HCC). Pivotal factors include chronic infections due to hepatitis B (HBV) and hepatitis C (HCV) viruses, which frequently result in cirrhosis and an increased risk of malignancy. In recent years, the metabolic dysfunction-associated steatotic liver disease (MASLD) and its progression in metabolic dysfunction-associated steatohepatitis (MASH) have emerged as a common cause of HCC [4]. Despite continuous developments in treatment options, the prognosis for many patients remains poor. Currently, there are limited adjuvant or neoadjuvant treatment options available for a systemic approach [5].

Apart from its neoplastic cells, a tumor consists also of its surrounding tissue and various cell types. A particular focus on the immune cells within this environment is referred to as the tumor immune microenvironment (TIME) [6]. Each type of cancer evolves its own unique TIME, which affects numerous processes related to tumor progression and malignancy [7]. Among the infiltrating immune cells, tumor-associated macrophages (TAMs) are particularly prominent. Under *in vitro* conditions, different macrophage populations can be distinguished, with the M1- and M2-like types being of special interest. However, this simplified classification does not adequately represent the complexity observed *in vivo*. Instead, macrophages exist along a dynamic spectrum rather than merely a dichotomy of M1- and M2-like phenotypes [8]. Nevertheless, the M1-like phenotype is considered to have more anti-tumorigenic and pro-inflammatory properties, while the M2-like phenotype—characterized by its anti-inflammatory and tumor-promoting features—plays a supportive role in tumor development [9].

One factor contributing to tumor progression is oxidative stress. The key transcriptional regulator in the defense against oxidative stress is the transcription factor nuclear factor erythroid 2-related factor 2 (NFE2L2 or Nrf2). Although Nrf2 is constantly expressed, it undergoes rapid degradation under basal conditions through the Kelch-like ECH-associated protein 1 (KEAP1)–E3 ubiquitin ligase complex. The presence of oxidants alters Keap1’s conformation, thereby liberating Nrf2 and allowing it to translocate into the nucleus, where it induces the transcription of various genes [10]. Furthermore, Nrf2 promotes the expression of genes involved in tissue repair and regeneration.

In case of cancer, this essential defense system can be hijacked by tumor cells. Initially, during early tumor development, the Nrf2 system acts to counteract oxidative damage, reduce inflammation, and limit tumor initiation. However, if a tumor originates and progresses, Nrf2 appears to be taken over by the tumor cells and begins to function as an oncogenic mediator [11]. This shift is likely linked to its induction of genes coding for cytoprotective factors within the tumor. Recent evidence suggests that Nrf2 activity influences changes in macrophage polarization throughout tumor progression while simultaneously favoring the emergence of the tumor-promoting M2-like phenotype. For instance, a study conducted in 2023 utilizing a mouse model for colon adenocarcinoma demonstrated that hemorrhage-activated Nrf2 in TAMs can impact both tumor growth and sensitivity to antibody immunotherapy [12]. Nonetheless, our understanding of Nrf2’s role remains not fully understood regarding how it affects polarization change and its specific contribution to altered adaptive immune responses. For this reason, investigating interactions between the different cell types of TIME with the tumor is crucial for advancing cancer immunotherapies.

Traditionally, classical *in vitro* analysis of tumor cells was used in monolayer cell culture in the past. Recent advancements have led to the establishment of three-dimensional (3D) tumor cell culture models [13,14]. These models offer various advantages over conventional 2D cell culture, including more realistic cell–cell contact, formation of extracellular matrix, and more representative gene expression profiles comparable to solid tumors due to their 3D structure [15,16]. Furthermore, combining various TIME cell types within a 3D multicellular tumor spheroid model is possible. These aspects of the model enable more comprehensive studies to be conducted, such as investigating the ability of cancer cells to resist certain drugs. With an accurate *in vitro* model, initial animal studies could potentially be minimized according to the 3R principle (Replacement, Reduction, Refinement), enabling researchers to address primary questions more effectively [17]. Despite these advancements, there are still very few HCC spheroid models [18,19,20], especially those examining Nrf2’s role in metastatic potential, development, and TIME [21].

Therefore, we tested various methods to co-culture these cells of interest and developed a single- as well as a multicellular 3D HCC spheroid model to better analyze these interactions. In this study, we focused on optimizing protocols for generating reliable and reproducible spheroids as well as multicellular tumor models using a murine non-alcoholic steatohepatitis (NASH)-HCC cell line (N-HCC25), cells, and bone marrow-derived macrophages (BMDMs). To investigate the role of Nrf2 in tumor cells, we generated CRISPR/Cas9-mediated Nrf2 knockout (KO) and Keap1-KO cell lines. The ability of these modified cell lines to form spheroids was also assessed. We successfully implemented several methods to analyze the formed spheroids, including RT-qPCR, invasion assays, and ELISA. Additionally, we investigated how varying levels of Nrf2 in BMDMs influence the TIME. This model will enable upcoming studies to more accurately address the impact of Nrf2 on both the tumor and TIME.

## 2. Materials and Methods

### 2.1. Cell Culture

#### 2.1.1. N-HCC25 Cell Line

The N-HCC25 cell line was previously established by our group [22]. N-HCC25 were cultivated with Dulbecco’s Modified Eagle’s Medium (DMEM) high glucose containing 1% penicillin/streptomycin and 10% fetal bovine serum (FBS), hereinafter referred to as normal medium. Cells were maintained at 37 °C with 5% CO_2_ in a humidified incubator. The used cell culture plastics were coated with collagen A (0.1 mg/mL). The detachment of the cells was conducted with a short incubation with trypsin/EDTA for 5 min at 37 °C.

#### 2.1.2. Primary Murine Macrophage Isolation

Bone marrow-derived macrophages (BMDMs) were isolated from Vav-iCre∷Nrf2-loxP mice (referred to as Nrf2-KO), while Cre-negative mice of the same strain were used as phenotypic WT controls. This strain was generated by cross-breeding the two parental mouse strains, which were obtained from The Jackson Laboratory (Bar Harbor, ME, USA): Vav-iCre (JaxLab #008610) and Nrf2-loxP (JaxLab #025433). The mice were housed under specific pathogen-free conditions with a 12 h light/dark cycle and were given free access to food and water in our animal facility. Sampling of the bone marrow was conducted postmortem in accordance with the German legislation governing animal studies and the European animal protection directive 2010/63/EU. Bone marrow was isolated from the femora and tibiae of the mice as described by Zhang et al. [23]. If more cells were needed, we also isolated the bone marrow from the humeri. The cells were cultivated in 25 mL of pre-warmed L929-conditioned medium (LCM) 20 medium (normal medium + 20% LCM). On the third day, 15 mL of fresh LCM20 was added. On the sixth day, the medium was replaced by fresh LCM25 medium (normal medium + 25% LCM). Cells were incubated at 37 °C in a 5% CO_2_ atmosphere.

#### 2.1.3. CRISPR/Cas9-Mediated KO of Nrf2 and Keap1

For the generation of N-HCC25-KO cells, the clustered regularly interspaced short palindromic repeats (CRISPR)/Cas9 system was used. The following sgRNA sequences were used to target the murine Keap1 (5′-GCGAGGAGATCGATGAGTAAC-3′) as well as a nontargeting scramble control (5′-GTTCCGGGCTAACAAGTCCT-3′). The sgRNA were cloned into the lentiCRISPR v2 vector (Addgene plasmid #: 52961; http://n2t.net/Addgene:52961; RRID:Addgene_52961; accessed 12 March 2026), which was generated in Feng Zhang’s lab [24]. This plasmid provides a puromycin cassette for later selection of stably transduced cells. For Nrf2-KO, the following sgRNA sequence was used to target the murine Nrf2 (5′-TTCAACCCGAAGCACGCTGAAGG-3′). A restriction–ligation approach was used to construct the recombinant plasmid vectors as described in the provided cloning protocol. The ligated plasmid was introduced into competent *E. coli* DH5 alpha cells via heat shock, which were plated on agar plates afterwards. Successfully transformed *E. coli* were selected with 100 ng/mL ampicillin. Colonies were picked and subcultured in 10 mL LB broth to amplify the plasmids for control sequencing. The plasmids were introduced into N-HCC25 cells via lentiviral transduction as described below. The use of puromycin ensured that only successfully transduced N-HCC25 cells were subcultured.

#### 2.1.4. Fluorescent Labeling of Cells

Fluorescent proteins were used to identify N-HCC25 cells as well as BMDMs within an MCT. Fluorescent labeling was achieved by lentiviral transduction of the cells with either pLVX-mCherry (Addgene #: 180646; http://n2t.net/Addgene:180646; RRID: Addgene_180646 [25]; accessed 12 March 2026; N-HCC25 cells) or pLVTHM (Addgene #: 180646; http://n2t.net/Addgene:12247; RRID: Addgene_12247 [26]; accessed 12 March 2026; BMDMs). Viral transduction was conducted as described in the following. MCTs were transferred to µ-Slide 8-well with glass bottoms to be imaged in a Keyence BZ-9000 (Keyence Deutschland GmbH, Frankfurt a. M., Germany)

#### 2.1.5. Production of Lentiviral Particles and Transduction of Target Cells

Lentiviral particles were generated in HEK293T cells. Transfection was performed using the jetPEI^®^ DNA Transfection reagent (Peqlab, Erlangen, Germany). A second-generation lentiviral plasmid system was employed, comprising the lentiviral transfer vector (lentiCRISPRv2-sgNTC, lentiCRISPRv2-sgKeap1, lentiCRISPRv2-sgNrf2, pLVX-mCherry, or pLVTHM), the packaging vector (psPAX2: a gift from Didier Trono, School of Life Sciences, Ecole Polytechnique Federale de Lausanne, Laboratory of Virology and Genetics, Lausanne, Switzerland; Addgene #: 12260; http://n2t.net/Addgene:12260; RRID:Addgene_12260; accessed 12 March 2026), and the envelope protein (pMD2.G: a gift from Didier Trono, Addgene #: 12259; http://n2t.net/Addgene:12259; RRID:Addgene_12259; accessed 12 March 2026). Transduction was performed in standard culture medium (2 mL) with 5 × 10^5^ target cells (N-HCC25 or BMDMs, respectively) per well of a 6-well plate. Viral content with a multiplicity of infection of 10 was added to the cells directly after seeding. After a 16 h transduction period, the cells were washed with phosphate-buffered saline (PBS), and the cell culture medium was replaced. Following successful selection with puromycin, the cells were subjected to the experimental procedures.

### 2.2. Experimental In Vitro Models (See Figure S1)

#### Conditioned Media = Tumor Spheroid and Macrophages CM Model (Figure S1A)

In this co-culturing approach, BMDMs and N-HCC25 tumor spheroids were cultured with different pre-conditioned media. Macrophages were cultured for 24 h either in 50% (*v*/*v*) tumor spheroid supernatant to mimic a tumor-like situation for the macrophages (T), or in normal cell culture media, whereby they remained naive macrophages (C). Tumor spheroids were subsequently cultured in 50% (*v*/*v*) medium of TAM-like macrophages (TCM) or 50% (*v*/*v*) medium of naïve macrophages (MCM) for 24 h.

### 2.3. Co-Culture Models

#### 2.3.1. Transwell = Spheroid in Well and BMDMs in Transwell (Figure S1B)

In this approach, ThinCert^®^ Cell Culture Inserts for 24-well plates and a pore size of 0.4 μm (Greiner Bio-one, Kremsmünster, Austria) were used to bring tumor cells and BMDMs in close distance without direct physical cell–cell contact. N-HCC25 tumor spheroids and BMDMs were cultivated as described above. After 6 days, 10^4^ BMDMs were transferred into a ThinCert^®^ 24-well insert and cultivated in LCM25 medium overnight. In each well, 12 spheroids were placed onto the collagen A-coated 24-well bottom. The BMDM containing ThinCert^®^ insert was placed into the well above the spheroids, and the co-culture was incubated in normal medium for 2 days.

#### 2.3.2. Transwell Tower = Spheroids Inside the Transwell, BMDMs on the Bottom (Figure S1C)

In another co-culture approach, a modified version of the double-sided co-culturing model described by Kang and colleagues was applied [27]. ThinCert^®^ Cell Culture Inserts were used to bring tumor spheroids and BMDMs into close proximity and allow direct cell–cell contact. A 3D printed ring, same size as the ThinCert^®^ inserts, was attached to the bottom side with Parafilm^®^, with a lid containing a star-shaped spacer for oxygen supply but maintaining sterile conditions (Figure S6A–C: 3D prints provided by Gunnar Böttcher, Institute of Automotive Engineering, RWTH Aachen University). The 3D-printed modifications were sterilized with 96% ethanol and a 30 min treatment with UV-light. The N-HCC25 tumor spheroids and BMDMs were cultivated as described. To allow BMDMs to attach to the bottom surface of the ThinCert^®^ membrane, the ThinCert^®^ insert was mounted upside down into a custom 3D-printed ring, and the underside of the ThinCert^®^ insert’s membrane was coated with collagen A. Cell suspension containing 2 × 10^5^ BMDMs was then applied to this bottom membrane surface. BMDMs were cultivated for 24 h for proper adherence to the lower membrane (Figure S6D). The next day, the construction was disassembled, and the ThinCert^®^ inserts with the BMDMs attached to the underside of the membrane were placed in a 12-well plate. Afterwards, 12 N-HCC25 spheroids were placed inside each insert. Cells were co-cultured using normal medium for 48 h.

#### 2.3.3. Spheroid and Multicellular Tumor Spheroid (MCT) Model (Figure S1D)

Spheroids were formed through a scaffold-free system, using the Nunclon^TM^ Sphera^TM^ 96-well plates (Thermo Fisher Scientific, Waltham, MA, USA). Cells were seeded into the plate with an initial amount of 7500 cells per well, in a total volume of 200 µL normal medium. The plate was briefly centrifuged at 1000× *g* to prevent the formation of multiple spheroids per well. Cultivation was conducted in a humidified atmosphere, 5% CO_2,_ and 37 °C. MCTs were similarly prepared. 7500 N-HCC25 cells and 7500 BMDMs were mixed before seeding into the Nunclon^TM^ Sphera^TM^ 96-well plate. Cultivation was under the same standards as mentioned above.

## 3. Readouts

### 3.1. Spheroid Morphology

Spheroid formation was monitored over a period of six days using the Incucyte^®^SX5 (Sartorius, Göttingen, Germany) system. Each well was automatically photographed in a 4 h interval over six days. Afterwards, spheroid formation and morphology were analyzed with the integrated Incucyte software version 2024A.

### 3.2. Viability Assay

To investigate the viability of N-HCC25 cells in our spheroid model and to examine the influence of an Nrf2- or Keap1-KO on viability, the CellTiter-Glo^®^ 3D Cell Viability Assay (#G9681, Promega, Madison, WI, USA) was performed. The assay was conducted according to the manufacturer’s instructions.

### 3.3. Live/Dead Assay

To morphologically investigate the viability of BMDMs and to examine the influence of an Nrf2-KO on viability, the LIVE/DEAD^®^ Cell Imaging Kit (#R37601, Thermo Fisher Scientific, Waltham, MA, USA) was used. For the assay, 2 × 10^5^ BMDMs were seeded in a 6-well plate and incubated for 48 h. The assay was conducted according to the manufacturer’s instructions, and images were captured with the Keyence BZ-9000.

### 3.4. CellTiter Blue Assay

To investigate the viability of the BMDMs and to examine the influence of an Nrf2-KO on viability, the CellTiter-Blue^®^ Cell Viability Assay (#G8081, Promega, Madison, WI, USA) was performed. For the CellTiter-Blue assay, 2 × 10^4^ cells were seeded in a 96-well plate and incubated for 48 h. The assay was conducted according to the manufacturer’s instructions, and viability was detected with the Tecan Infinite M200 PRO (Tecan Trading AG, Männedorf, Switzerland).

### 3.5. Invasions Assay

In order to analyze the migratory behavior of the tumor cells in the tumor as well as MCT, a matrix-based invasion assay was performed. On day 5 of spheroid formation, 100 µL of supernatant was discarded and replaced with 100 µL Corning^®^ Matrigel^®^ Matrix (#356237, Corning, NY, USA), thereby embedding the whole spheroid in a final Matrigel concentration of 4.5 mg/mL (50%). All required pipetting steps were performed with pre-chilled pipette tips and on a cold pack to prevent early polymerization. Invasion of the tumor cells was monitored for a period of 24 h, with a schedule of repeated photography at 0, 1, 3, 6, 12, and 24 h. Invasion of the MCT was monitored after 24 h. For quantitative analysis, invasion assay images were segmented using the Trainable Weka Segmentation plugin in Fiji [28]. The supervised classifier based on a Fast Random Forest model was trained on 20 representative images. Three segmentation classes were defined: spheroid core, invasive protrusions (“spikes”), and background. For each class, approximately 5–6 manually annotated traces per image were provided as training data. Classifier training was performed using a predefined feature set including Gaussian blur, Difference of Gaussian, Hessian, and Sobel filters, membrane projections, and first- and second-order statistical features (mean and variance). The random forest seed was fixed (seed = 1209041686) to ensure reproducibility. The trained classifier was subsequently applied to all invasion assay images using batch processing. A representative example of the defined segmentation classes and quantified areas is shown in Figure S2.

### 3.6. ELISA

Cell culture supernatant was harvested after six days of spheroid formation for Enzyme-linked immunosorbent assay (ELISA). The Mouse VEGF DuoSet^®^ ELISA kit (#DY493, R&D Systems, Minneapolis, MN, USA) was used according to the manufacturer’s prescribed protocol; 100 µL of undiluted supernatant was used. The plate was analyzed with the Infinite M200 Microplate Reader (Tecan Life Sciences, Männedorf, Switzerland).

### 3.7. Cryo-Sectioning and IF Staining of Spheroids

Eight spheroids were pooled in a 1.5 mL reaction tube and rinsed with 500 µL PBS. Spheroids were fixed in 4% formalin for 30 min at RT, followed by three washing steps with 500 µL PBS. For cryoprotection, samples were incubated sequentially in 10% and 20% sucrose for 30 min each at RT and subsequently in 30% sucrose overnight at 4 °C. Spheroids were embedded in Tissue-Tek^®^ O.C.T. (#4583, Sakura Finetek USA, Torrance, CA, USA) compound, cryosectioned at a thickness of 8 µm using a cryostat (Leica DM3050 Cryostat, Leica Microsystems GmbH, Wetzlar, Germany) and mounted on SUPERFROST^®^ PLUS slides (Gerhard Menzel GmbH, Leverkusen, Germany). For immunofluorescence staining, cryosections were thawed for 30 min at RT and rehydrated for 10 min in 0.1% Triton X-100 in PBS. Sections were washed with PBS for 5 min and blocked for 30 min at RT in the dark using 5% normal goat serum (NGS). Afterwards, the rabbit anti-mouse Iba1 antibody (#019-1941, Wako, Osaka, Japan; 1:200 in 5% NGS) was incubated for 1 h, at RT in the dark, followed by three 5 min PBS washes. Sections were incubated with donkey anti-rabbit Alexa Fluor 488 (#A21206, Thermo Fisher Scientific, Waltham, MA, USA) secondary antibody (1:200 in 5% NGS) for 1 h at RT in the dark and washed three times with PBS for 5 min. Nuclear counterstaining was carried out using bisbenzimide (1:10,000 in PBS) for 10 min at RT in the dark. After final washing steps with PBS and sterile H_2_O (5 min each), sections were mounted with ImmuMount™. Samples were imaged after 24 h using the BZ-9000 fluorescence microscope.

### 3.8. Flow Cytometry

For flow cytometry, 10 spheroids were pooled from each group. Therefore, 150 µL supernatant per well was removed, and the spheroids were pooled in a 1.5 mL reaction tube. To dissociate the MCT-spheroids, 800 µL of Accutase per reaction tube was used, and the spheroids were incubated for 10 min at 37 °C and 1000 rpm. Next, the spheroids were resuspended 20 times for further dissociation. The macrophage surface marker CD11b was stained using VioGreen anti-CD11b (#130-113-811, Miltenyi Biotec, Bergisch Gladbach, Germany); REA Control-VioGreen was used as an isotype control (#130-113-456, Miltenyi Biotec, Bergisch Gladbach, Germany). Antibodies were diluted 1:50 in staining buffer (0.5% BSA, 2 mM EDTA in PBS). Therefore, cells were resuspended in 50 μL antibody solution and incubated for 10 min at 4 °C protected from light. Cells were then washed with 1 mL staining buffer for 10 min and centrifuged (300 × *g*, 5 min, 4 °C). Pellets were resuspended in 1 mL cold Fixation/Permeabilization solution (FoxP3 Staining Buffer Set, #00-5523-00, Thermo Fisher Scientific, Waltham, MA, USA) and incubated for 30 min at 4 °C. Subsequently, cells were washed once with 1 mL staining buffer and once with 500 μL Permeabilization buffer (FoxP3 Staining Buffer Set), then resuspended in 400 μL PBS for acquisition. The samples were analyzed with a BD LSRFortessa™ flow cytometer according to the manufacturer’s instructions. The flow cytometry data were analyzed using FlowJo 10.4.2 (Waters Biosciences, Ashland, OR, USA). In order to exclude necrotic or damaged cells or cell debris from the analysis, viable cells were first gated using FSC-A vs. SSC-A plots before being examined for CD11b expression. The gating strategy is shown in Figure S3.

### 3.9. RNA Isolation, cDNA Synthesis, and RT-qPCR

RT-(q)PCR studies were performed according to the MIQE guidelines [29]. Total RNA was isolated from N-HCC25 in 2D cell culture as well as 3D culture from diverse passages. For each 3D culture RNA sample, 24 spheroids of the same experimental group were pooled as one biological replicate. RNA-Solv^®^ reagent (#R6830-02, Omega Bio-Tek, Norcross, GA, USA) was used according to the manufacturer’s instructions, but only 0.5 mL of RNA-Solv^®^ was used for the spheroids. Concentration and purity were determined spectrophotometrically using the NanoDrop^®^ND-1000 (Thermo Fisher Scientific, Waltham, MA, USA) device. MOPS buffered denaturing RNA gel electrophoresis (28S/18S rRNA ratio) was used to ensure sufficient RNA integrity. One µg of total RNA was reverse transcribed afterwards. Ambion DNAse I (#AM2222, Thermo Fisher Scientific, Waltham, MA, USA) was used to digest potential gDNA contamination in the RNA samples. DNase treatment was stopped with EDTA [5 µM]. For cDNA synthesis, mixed priming with oligo(dT)18 and random hexamer (molar ratio of 3:1) together with Maxima Reverse Transcriptase (#SO132, #SO142, #EP0743, Thermo Fisher Scientific, Waltham, MA, USA) was utilized. Real-time PCR was performed on an ABI StepOne Plus or QuantStudio 3 system using 15 ng cDNA per reaction. Amplification was carried out with either Power SYBR™ Green PCR Master Mix (#4367659, Thermo Fisher Scientific, Waltham, MA, USA) or PowerTrack™ SYBR Green Master Mix (#A46111, Thermo Fisher Scientific, Waltham, MA, USA). For data normalization, twelve potential reference targets and five representative samples per experimental group were evaluated for the establishment of a reference gene index using geNorm calculations (Biogazelle qbase+ 3.4 software, Zwijnaarde, Belgium). For the different qPCR studies, a combination of various reference genes was found to be the most suitable for data normalization. Analysis of the transwell constructs: Cullin 4A (*Cul4a*) and tyrosine 3-monooxygenase/tryptophan 5-monooxygenase activation protein zeta polypeptide (*Ywhaz*), and for the analysis of the MCT: *Cul4a*, eukaryotic translation elongation factor 2 (*Eef2*), and hypoxanthine guanine phosphoribosyl transferase (*Hprt*). The use of inter-run calibrators ensured the correction of potential inter-run variations. All experiments were performed with primer-specific annealing temperatures (Table 1 and Table 2: T_A_) and a standard protocol (Table S1). Through TAE-buffered DNA agarose gel electrophoresis and a melt-curve analysis, primer specificity was determined (Table 1: T_M_ and amplicon length).

The PCR amplification efficiency was calculated with LinRegPCR software version 2017.0 (Heart Failure Research Center, Amsterdam, The Netherlands) [30]. The software qBase Plus 3.4 (Biogazelle, Zwijnaarde, Belgium) was used to calculate the relative fold-change in gene expression in accordance with the efficiency-corrected ΔΔCq method.

### 3.10. Protein Isolation and Western Blot

For the isolation of total protein from N-HCC25 cells, 5 × 10^6^ cells were seeded on 10 cm dishes the day before the isolation. Cells were washed with ice-cold PBS before ice-cold RIPA buffer, including cOmplete™ Mini protease inhibitor cocktail (#11836170001; Roche, Basel, Switzerland) was added to the dish. Lysis was intensified by mechanical force in a Precellys^®^ homogenizer (Bertin Technologis, Montigny-le-Bretonneux, France) for 20 s at 5000 rpm to obtain total protein samples. The Pierce™ BCA Protein Assay Kit was used to determine protein concentrations as described by the manufacturer (#A55864; Thermo Fisher Scientific Inc., Waltham, MA, USA). An amount of 10 µg of total protein was heat-denaturized in NuPage™ LDS sample buffer (#NP0007; Thermo Fisher Scientific Inc., Waltham, MA, USA) containing dithiothreitol before SDS-PAGE separation using 10% polyacrylamide gels. Proteins were transferred onto a PVDF membrane by semi-dry electro-blotting (Trans-Blot^®^ Turbo™, BioRad, Hercules, CA, USA). After blocking in 5% milk powder in TBS T for 1 h at RT, the membrane was incubated with the primary antibody Nqo1 [EPR3309] (1:1000 in 5% TBS-T, #ab80588; Lot.: GR33174; monoclonal rabbit; abcam Ltd., Cambridge, UK) overnight at 4 °C. Afterwards the HRP-conjugated anti-rabbit immunoglobulin G secondary antibody (1:20,000 in TBS-T; #7074; CST, Leiden, The Netherlands) was applied for 1 h at RT under continuous gentle shaking. Immunoreactivity was visualized using the chemiluminescence reagent Immobilon™ Western (#WBKLS; Millipore, Darmstadt, Germany) as recommended by the manufacturer. Luminescence signals were detected using a Fusion Solo imager (Vilber, Collégien, France). The antibody complexes were removed with Roti^®^ Free Stripping Buffer (#0083.1; Carl Roth, Karlsruhe, Germany) for 15 min at 60 °C before the membrane was blocked again, and the primary antibody α-Tubulin clone B-7 (1:1000 in 5% milk powder TBS-T; #sc-5286; monoclonal mouse; Santa Cruz Biotechnology Inc., Dallas, TX, USA) was applied at 4 °C overnight. Subsequently, the HRP-conjugated anti-mouse immunoglobulin G secondary antibody (1:20,000; #A2304; Sigma Aldrich, Frankfurt, Germany) was applied for 1 h at RT before immunoreactivity was analyzed as mentioned above. For densitometry, the Quantity One^®^ software version 4.6.9 (BioRad, Hercules, CA, USA) was used. The ratio between band intensities of Nqo1 and the corresponding band of α-Tubulin was normalized to the non-mammalian target control (NTC) group.

### 3.11. Statistics

Variance homogeneity (Bartlett’s test) and normal distribution (Shapiro–Wilk test) were checked. If necessary, data were log10 or Box–Cox transformed to achieve homoscedasticity. For parametric data, ANOVA-based tests were used to analyze the data. The tests used in each experiment are indicated in the corresponding figure legends. Data are represented as mean + standard deviation (SD). All statistical analyses were executed with BioMedStatX version 1.0.1 [31] and GraphPad Prism 10 (GraphPad, San Diego, CA, USA).

## 4. Results

**Figure 1:**
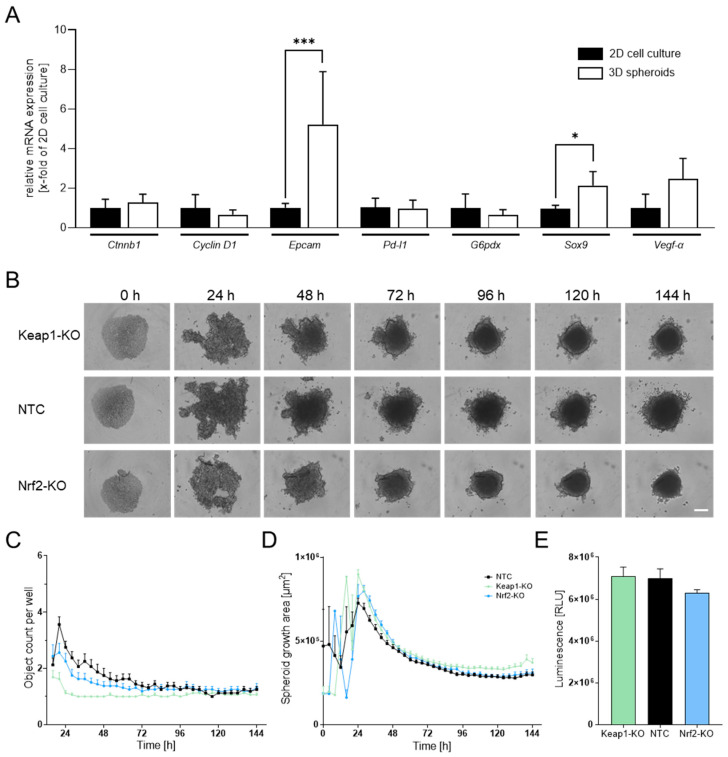
N-HCC25 3D spheroids display enhanced tumor-associated characteristics compared to 2D culture. (**A**) RT-qPCR analysis of tumor-associated markers in N-HCC25 cells cultured under 2D or 3D conditions. For 2D, 3.5 × 10^5^ cells were used per biological replicate; for 3D, 24 spheroids were pooled per replicate. Gene expression was normalized using the reference genes *Cul4a* and *Ywhaz*. Data represent mean + SD, *n* = 4–6. Student’s *t*-test was used for statistical analysis. (**B**) Time-course imaging of spheroid formation from nontargeting scramble control (NTC), Nrf2-KO, and Keap1-KO N-HCC25 cells over 6 days using Incucyte^®^ live-cell imaging; representative images are shown. Scale bar = 250 µm. (**C**) Quantification of spheroid object count per well over time. (**D**) Spheroid growth area measured across the 6-day period. (**E**) Viability of NTC and KO spheroids assessed using the CellTiter-Glo^®^ 3D assay. One-way ANOVA was used for statistical analysis. Data represent mean + SEM, *n* = 8. Statistical significance is indicated as * *p* < 0.05, *** *p* < 0.005.

To determine whether 3D spheroid culture provides advantages over conventional 2D conditions for N-HCC25 cells, we first performed an RT-qPCR analysis of tumor-associated marker genes. N-HCC25 spheroids showed significantly higher expression of *Epcam* and *Sox9* compared to 2D culture. Both genes are associated with cancer stemness and tumor recurrence. These findings support that 3D spheroids display more pronounced tumor-related characteristics than 2D cultures (Figure 1A).

Next, we generated Nrf2- and Keap1-KO N-HCC25 cell lines using CRISPR/Cas9 and validated the modifications by RT-qPCR and Western blot. Keap1 transcript levels were strongly reduced in the Keap1-KO line, while gene expression of the Nrf2 target gene Nqo1 was significantly decreased in the Nrf2-KO line, confirming efficient disruption of Nrf2 signaling. As expected, Nqo1 expression was markedly elevated in the Keap1-KO line, demonstrating the inhibitory role of Keap1 on Nrf2 activity (Figure S4A). This was further confirmed on the protein level as Nqo1 protein expression was significantly reduced in Nrf2-KO while extensively increased in Keap1-KO N-HCC25 cells (Figure S4B,C). To further characterize the KO cell lines, we evaluated spheroid morphology and viability. Spheroid formation was monitored over six days using live-cell imaging (Figure 1B). All groups consistently formed a single spheroid per well (Figure 1C). The mean spheroid area stabilized at approximately 3 × 10^5^ µm^2^ across conditions, with Keap1-KO spheroids showing a slightly larger, but not statistically significant, area (Figure 1D). Viability analysis revealed no significant differences between groups, although Nrf2-KO spheroids displayed a non-significant trend toward reduced viability compared to nontargeting scramble control (NTC) spheroids (Figure 1E).

**Figure 2:**
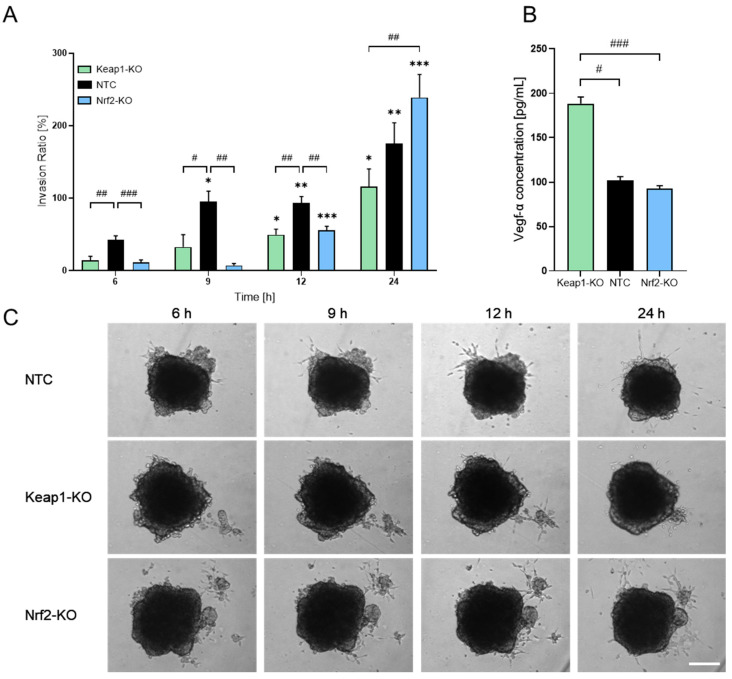
N-HCC25 spheroids exhibit altered invasive behavior and Vegf-α secretion. (**A**) On day 5 of spheroid formation, N-HCC25 spheroids (NTC, Nrf2-KO, Keap1-KO) were embedded in 50% Matrigel^®^. Invasive outgrowth was monitored for 24 h, with quantification starting after a 6 h equilibration phase. The invasion area was analyzed using the Trainable Weka Segmentation plugin in Fiji. Data represent mean + SEM, *n* = 8. Mixed-model analysis was used to assess genotype- and time-dependent effects. Statistical significance for time-dependent changes within each genotype is indicated as * *p* < 0.05, ** *p* < 0.01, *** *p* < 0.005 compared to the same genotype at 6 h. Statistical significance between genotypes at each time point is indicated as # *p* < 0.05, ## *p* < 0.01, and ### *p* < 0.005. (**B**) Vegf-α concentration in spheroid supernatant after 6 days of culture. For each replicate, duplicates of 100 µL undiluted supernatant were analyzed by ELISA. Data represent mean + SEM, *n* = 8. For statistical analysis, the Kruskal–Wallis test was performed with a subsequent Dunn test. Significance is indicated as # *p* < 0.05, ### *p* < 0.005. (**C**) Representative images of the invasion assay illustrating spheroid morphology and invasive protrusions. Scale bar = 250 µm.

**Figure 3:**
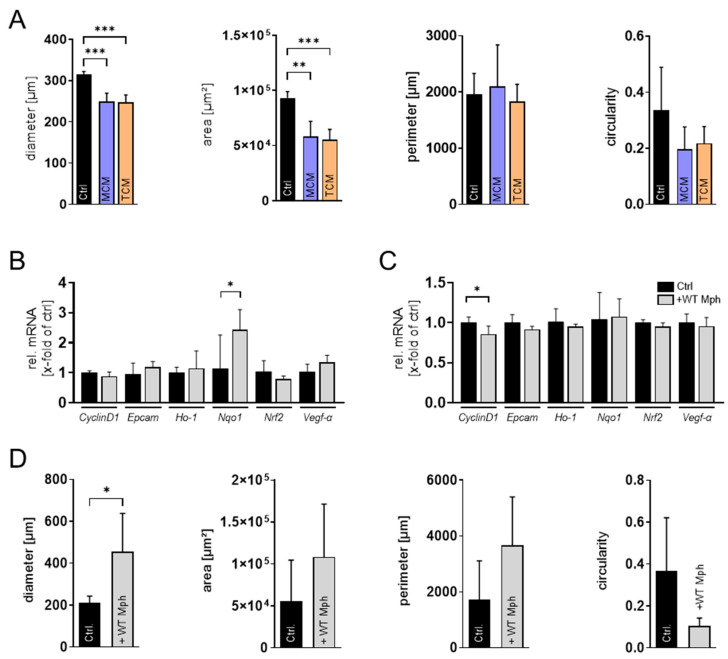
N-HCC25 spheroids respond differentially to four co-culture approaches with macrophages. (**A**) N-HCC25 spheroids were treated for 6 days with conditioned medium from naïve macrophages (MCM) or TAM-like macrophages (TCM) and normal medium (Ctrl). Morphology was quantified using ImageJ version 1.54p. Statistical analysis was performed with a one-way ANOVA and subsequent Tukey’s HSD post hoc test. Data represent mean + SD, *n* = 4–6. (**B**,**C**) RT-qPCR was conducted after 48 h of co-culture in the transwell system. Gene expression was normalized to *Cul4a* and *Ywhaz*. Statistical analysis was performed with Student’s *t*-test. Data represent mean + SD, *n* = 5–6. (**D**) Multicellular tumor spheroid morphology was assessed after 6 days of co-culture with WT macrophages using ImageJ version 1.54p. Statistical analysis was performed with Student’s *t*-test. Data represent mean + SD, *n* = 5–6. Significance is indicated as * *p* < 0.05, ** *p* < 0.01, *** *p* < 0.005.

With the established 3D spheroid model, we next analyzed additional parameters of tumor cell behavior. First, we performed an invasion assay to assess the invasive potential of the different N-HCC25 cell lines using the trainable Weka Segmentation plugin in Fiji (Figure 2A,C and Figure S2). The invasion ratio increased significantly over time in NTC tumor cells compared to the initial 6 h time point. In contrast, Keap1- as well as Nrf2-KO did not show a significant increase in invasion before 12 h after adding Matrigel^®^. Moreover, these cells also showed significantly lower invasion ratios during the first three measurement time points (6 h, 9 h, and 12 h) compared to the NTC cells. Interestingly, after 24 h, Nrf2-KO tumor cells exceeded the invasion of NTC cells, while Keap1-KO cells still exhibited reduced invasive behavior compared to NTC cells. In parallel, we examined Vegf-a secretion by conducting an ELISA on spheroid supernatants. Keap1-KO spheroids displayed significantly increased Vegf-α levels compared to NTC spheroids and Nrf2 (Figure 2B). As a subsequent step, we sought to incorporate macrophages into the system to better mimic the tumor immune microenvironment. For this purpose, we implemented four different co-culture approaches involving conditioned media, transwell systems, and direct-contact settings (Figure S1A–D). Initially, we analyzed cell viability as well as the proportions of living cells in our BMDM cultures. Live/dead staining revealed that Nrf2-KO BMDM cultures have a slightly decreased amount of living cells compared to WT BMDM cultures (Figure S5A,B). The CTB assay further confirmed that Nrf2-KO BMDMs exhibit slightly reduced cell viability compared to WT BMDMs (Figure S5C). In the first co-culture approach, we exposed N-HCC25 spheroids to conditioned media derived either from naïve macrophages (MCM) or TAM-like macrophages (TCM) (Figure S1A). Treatment with conditioned medium did reveal significant reductions in diameter and area compared to the control, but no significant effects were observed for perimeter and circularity, and between treatment with MCM and TCM (Figure 3A). In the second approach, we brought tumor spheroids and macrophages into closer proximity without enabling direct physical contact. Spheroids were cultured on the bottom of a 24-well plate, while BMDMs were placed in a ThinCert^®^ insert positioned above (Figure S1B). Only for the *Nqo1* spheroid gene expression, RT-qPCR analysis revealed significant upregulation (Figure 3B). In the third approach, we aimed to enable limited physical interaction by positioning macrophages on the underside of the ThinCert^®^ membrane while placing spheroids inside the insert (Figures S1C and S6A–D). This configuration allows potential contact between macrophage cell extensions and tumor spheroids through the membrane pores. RT-qPCR analysis of spheroids in this setup revealed no major alterations, with the exception of a significant reduction in CyclinD1 expression compared to the control (Figure 3C).

**Figure 4:**
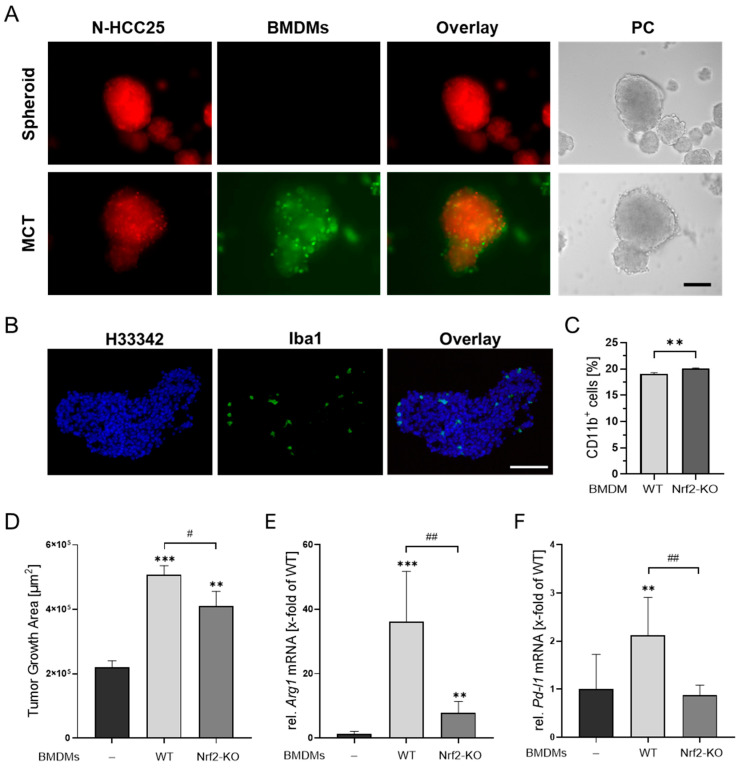
MCT tumor spheroids provide a suitable *in vitro* model to study N-HCC25 tumor–macrophage interactions. (**A**) Visualization of cellular composition in MCT using mCherry-expressing N-HCC25 tumor cells (red) and eGFP-labeled macrophages (green). Representative fluorescence and phase contrast (PC) images are shown. Scale bar = 250 µm. (**B**) Immunofluorescence staining of cryosectioned MCT (8 µm). Macrophages were detected using an Iba1 antibody (green), and nuclei were counterstained with Hoechst 33342 (blue). Scale bar: 250 µm. (**C**) Flow cytometric quantification of macrophage content in MCT. Ten spheroids per condition were pooled, as well as a cell-matching amount of 2D-cultured BMDMs, enzymatically dissociated, and stained for CD11b to determine the proportion of macrophages. data = mean + SD, *n* = 5. (**D**) Matrigel^®^-based assay of tumor and MCT on day 5 of spheroid formation. The invasive area was quantified 24 h after Matrigel^®^ addition using the Trainable Weka Segmentation plugin in Fiji (Figure S2). Data are presented as mean + SEM, *n* = 8. One-way ANOVA with Tukey’s HSD post hoc test. (**E**,**F**) Gene expression analysis of tumor and MCT after 6 days of spheroid formation. RT-qPCR data were normalized to *Cul4a, Eef2,* and *Hprt*. Data are presented as mean + SD, *n* = 5–6. Statistical analysis was performed using one-way ANOVA with Tukey’s HSD post hoc test. Statistical significance indicated ** *p* < 0.01, *** *p* < 0.005 compared to tumor spheroids and # *p* < 0.05, ## *p* < 0.01 for comparisons between MCT groups.

In the final step, we established multicellular tumor (MCT) spheroids by premixing N-HCC25 tumor cells with macrophages and allowing them to aggregate into a single spheroid. Morphological changes were observed and revealed significant enlarged diameter and a tendency in enlarged area and perimeter. The circularity tended to decrease when adding macrophages (Figure 3D). To verify the cellular composition of the MCTs, we performed flow cytometry and immunofluorescence analyses. Fluorescent reporters, with N-HCC25 cells expressing mCherry and macrophages labeled with eGFP, confirmed macrophage integration and migration into the MCT structure (Figure 4A), also confirmed by immunofluorescent macrophage-specific Iba1 staining (Figure 4B) and flow cytometry, subsequently quantified 19.1% WT and 20.1% Nrf2-KO macrophages within the MCTs (Figure 4C). Morphological analysis demonstrated a significantly increased tumor spheroid growth area in MCTs containing either WT or Nrf2-KO macrophages. However, MCTs with Nrf2-KO macrophages exhibited a significantly reduced invasive area compared to MCTs containing WT macrophages (Figure 4D).

To assess the influence of macrophage-intrinsic Nrf2 levels within the MCT, we performed RT-qPCR analyses of marker genes known for their immunosuppressive effect within the TIME. Expression of *Arg1*, a marker associated with anti-inflammatory macrophage phenotypes, was significantly elevated in MCTs containing WT macrophages compared to spheroids composed of N-HCC25 tumor cells alone. *Arg1* expression was also increased in MCTs containing Nrf2-KO macrophages. Nonetheless, the expression was significantly lower in MCTs with Nrf2-KO macrophages compared to those with WT macrophages (Figure 4E).

Programmed cell death-ligand 1 (*Pd-l1*), which can be expressed by both tumor cells and M2-like macrophages, was upregulated in MCTs containing WT macrophages relative to N-HCC25 spheroids alone, which was not the case for MCTs that contained Nrf2-KO macrophages (Figure 4F).

## 5. Discussion

In this study, we successfully established a reproducible MCT model by combining N-HCC25 cells and BMDMs to recreate the specific crosstalk between tumor cells and macrophages within the TIME. TAMs are among the most abundant immune cells in the HCC microenvironment and are recognized as key regulators of tumor progression and therapy response [32,33]. While 3D culture of tumor cells alone enhanced tumor stemness characteristics compared to 2D monolayers, our data indicate that the incorporation of macrophages was crucial to increase spheroid diameters, in line with the established concept that stromal and immune components critically shape tumor progression in HCC [34]. By utilizing CRISPR/Cas9-mediated gene editing and macrophage-specific knockout models, we demonstrated that Nrf2 signaling, both tumor-intrinsic as well as macrophage-intrinsic, plays a distinct role in shaping this interaction. However, although Nrf2-KO cells displayed reduced Vegfa expression, the matrigel-based invasion assays were conducted in a tumor-cell-only *in vitro* system lacking endothelial and stromal components, so the canonical angiogenic function of Vegfa might not be engaged and is unlikely to drive the observed migratory phenotype. We therefore interpret the increased migratory behavior of Nrf2 KO cells as a predominantly Vegfa-independent, cell-autonomous stress response, whereas the elevated Vegfa levels in Keap1-KO cells are expected to be more relevant for angiogenesis *in vivo* than for migration in vitro. Our most significant finding is that Nrf2 deficiency in macrophages attenuates their tumor-promoting phenotype, reducing both tumor growth and the expression of the immunosuppressive marker *Pd-l1* and the M2-macrophage marker *Arg1*. This identifies Nrf2 in TAMs as a potential driver of HCC progression, consistent with recent work showing that heme- and hemorrhage-driven Nrf2 activation in TAMs promotes tumor growth, invasion, and resistance to immunotherapy [12,35].

Our initial characterization confirmed that 3D spheroid culture more accurately reflects the physiological properties of solid tumors than conventional 2D cultures. We observed a significant upregulation of the stemness markers *Epcam* and *Sox9* in N-HCC25 spheroids compared to cells grown as monolayers. This aligns with the current understanding that the 3D architecture promotes enrichment of cancer stem cells (CSCs), particularly in HCC, where EpCAM and Sox9 are well-established stemness-associated markers [36]. More broadly, 3D spheroid models recapitulate nutrient and oxygen gradients, cell–cell and cell–matrix interactions, and drug penetration barriers that are absent in monolayer cultures, contributing to CSC enrichment and therapy resistance [37]. Additionally, 3D models of HCC have been shown to better capture drug responses and metabolic alterations compared to 2D cultures, underscoring their value for preclinical screening [38].

However, the tumor compartment alone is insufficient to model the metastatic cascade. Our comparison of different co-culture methods highlights the necessity of direct cell–cell contact. While conditioned media and transwell systems yielded only minor tumor characteristic effects, the direct integration of macrophages in the MCT model resulted in profound phenotypic, invasive outgrowth, and altered expression levels of important markers in the TIME. Similar observations have been made in multicellular HCC spheroid systems, where integration of stromal cells into the 3D structure more effectively induces invasive growth and chemoresistance than indirect co-culture or conditioned media alone [39]. This suggests that soluble factors or limited cell contact through the pores of ThinCert^®^ inserts alone are insufficient to fully recapitulate TAM–tumor crosstalk. It indicates that juxtacrine signaling or mechanical interactions are essential drivers of HCC progression. The importance of physical architecture and spatial organization for immune cell behavior and tumor plasticity is increasingly recognized in advanced 3D and organoid models of HCC [33,38].

Regarding tumor-intrinsic Nrf2 signaling, we simulated constitutive Nrf2 activation by generating Keap1-KO N-HCC25 cells. Interestingly, even if Keap1-KO did not significantly enhance invasive outgrowth, it led to a significant increase in Vegfa secretion. This supports the concept of the “dark side” of Nrf2, where cancer cells hijack this cytoprotective pathway to support angiogenesis, metabolic rewiring, and survival [11]. In the context of HCC, which is a highly vascularized tumor, Nrf2-mediated Vegfa upregulation could be a key mechanism connecting oxidative stress responses to neovascularization. We and others have shown that activation of the Nrf2/Ho-1 axis can increase Vegfa expression and promote angiogenesis, supporting a mechanistic link between Nrf2 signaling and pro-angiogenic programs [40,41].

A key strength of our study is that the established 3D MCT platform is sensitive enough to resolve Nrf2-dependent differences in macrophage polarization within a controlled tumor–TIME context. MCTs containing WT BMDMs showed pronounced invasive outgrowth and high expression of Arg1 and Pd-l1, markers typical of an M2-like, immunosuppressive phenotype. Strikingly, this effect was significantly blunted in MCTs containing Nrf2-KO macrophages, which showed reduced invasive capacity and lower expression of these markers. This implies that Nrf2 is required for TAMs to adopt their full tumor-promoting function. This observation is in line with studies identifying Nrf2 as a key regulator of macrophage polarization and anti-inflammatory/M2-like programs, often mediated via heme degradation and Ho-1 induction [34,42,43].

Despite these promising findings, our study has limitations that must be considered. First, our MCT model currently focuses solely on the interaction between tumor cells and BMDMs. The *in vivo* TIME is far more complex and includes vessels, critical adaptive immune cells, particularly T cells, stroma cells such as tumor-associated fibroblasts, as well as soluble factors and ECM composition [7]. In the context of immune cells, cytotoxic CD8+ T cells are the primary effectors of anti-tumor immunity in HCC, and their exclusion or functional exhaustion is a hallmark of immune escape and treatment resistance [44]. Since we observed Nrf2-dependent regulation of Pd-l1 in our model, incorporating T cells is a necessary next step to evaluate whether Nrf2 modulation in macrophages can effectively restore T cell–mediated killing and responsiveness to immune checkpoint blockade. Further, not all macrophages present in the culture are stably integrated into the spheroid core. Due to the relatively low cell number per spheroid, we had to pool multiple spheroids for molecular analyses, which precludes a single-spheroid-level resolution and may mask heterogeneity in macrophage distribution, activation state, and local Nrf2 activity. Complementary imaging-based approaches and single-spheroid transcriptomic profiling will be important to address this limitation. Moreover, it has already been shown that depending on their size, MCTs can contain hypoxic and apoptotic/necrotic regions that arise from oxygen and nutrient gradients. Hypoxia develops gradually as the spheroid size increases. Early studies showed that small micro-spheroids (<200 μm diameter) consist mainly of proliferating, normoxic cells, whereas further growth to ~200–300 μm produces a characteristic zonation with proliferative surface layers, normoxic quiescent cells in the middle, and hypoxic cells in the core [45]. Since hypoxia most probably has an impact on Nrf2 in our spheroid model, future studies need to implement analyses that take this into account.

In summary, we established a robust 3D MCT model that underscores the importance of Nrf2 in modulating the tumor–macrophage axis. We show that Nrf2 activation in tumor cells fuels angiogenesis, while Nrf2 in macrophages is critical for supporting tumor growth and immunosuppression, in line with the broader concept of oncogenic Nrf2 addiction and its immunoregulatory consequences in cancer. Future studies will aim to incorporate T cells into this model to further dissect how Nrf2 signaling within the innate immune compartment shapes the adaptive anti-tumor response in HCC.

## Figures and Tables

**Table 1:** Reference (Ref.) gene primer used in this study and its sequence. T_A_ = annealing temperature, T_M_ = melting temperature, bp = amplicon length, *Cul4a* = Cullin 4a, *Eef2* = eukaryotic translation elongation factor 2, *Hprt* = hypoxanthine guanine phosphoribosyl transferase, *Ywhaz* = tyrosine 3-monooxygenase/tryptophan 5-monooxygenase activation protein zeta polypeptide.

Ref. Genes	Sequence Accession Number	Direction	Sequence [5′-3′]	T_A_ [°C]	T_M_ [°C]	Amplicon Length [bp]
*Cul4a*	NM_001363450.1	Forward Reverse	TGATGCAGGACAGGGAGGTTC CCACACAGGCAATCAACGGT	59	79.4	123
*Eef2*	NM_007907.2	Forward Reverse	CACAATCAAATCCACCGCC ATGGCCTGGAGAGTCGATGA	60	80.7	122
*Hprt*	NM_013556.2	Forward Reverse	TCAGTCAACGGGGGACATAAA GGGGCTGTACTGCTTAACCAG	61	76.1	142
*Ywhaz*	NM_011740.3	Forward Reverse	GAAAAGTTCTTGATCCCCAATGC TGTGACTGGTCCACAATTCCTT	62	79.2	134

**Table 2:** Target gene primer used in this study and its sequence. T_A_ = annealing temperature, T_M_ = melting temperature, bp = amplicon length, *Arg1* = Arginase 1, *Ccnd1* = Cyclin D1, *Ctnnb1* = catenin beta 1, *Epcam* = epithelial cell adhesion molecule, *G6pdx* = Glucose-6-phosphate 1-dehydrogenase, *Keap1* = Kelch-like ECH-associated protein 1, *Nos2* = nitric oxide synthease 2, *Nqo1* = NADPH dehydrogenase quinone 1, *Nrf2* = nuclear factor erythroid 2-related factor 2, *Pd-l1* = programmed cell death-ligand 1, *Sox9* = SRY-box transcription factor 9, *Vegf-α* = vascular endothelial growth factor A.

Target Genes	Sequence Accession Number	Direction	Sequence [5′-3′]	T_A_ [°C]	T_M_ [°C]	bp
*Arg1*	NM_007482.3	Forward Reverse	AAGGACAGCCTCGAGGAGGGGTAG TGGACCTCTCCCACCACACCA	60.5	82	214
*Ccnd1*	NM_007631.2	Forward Reverse	GCGTACCCTGACACCAATCT CACAGACCTCCAGCATCCAG	60	83	160
*Ctnnb1*	NM_001165902.1	Forward Reverse	CTAGCTGGTGGACTGCAGAAA TTCAGCACTCTGCTTGTGGT	59	79.7	212
*Epcam*	NM_008532.2	Forward Reverse	CATTTGCTCCAAACTGGCGT TTGTTCTGGATCGCCCCTTC	60	79.5	125
*G6pdx*	NM_008062.3	Forward Reverse	GGGAAGAGTTGTACCAGGGTG TCTTCAGGTAGAAGGCCATCCC	59	80.1	142
*Keap1*	NM_001110305.1	Forward Reverse	GGCAGGACCAGTTGAACAGT CATAGCCTCCGAGGACGTAG	59	87.5	138
*Nos2*	NM_010927.4	Forward Reverse	ACCCTAAGAGTCACCAAAATGGC TTGATCCTCACATACTGTGCACG	60.5	82	118
*Nqo1*	NM_008706	Forward Reverse	AGAGAGTGCTCGTAGCAGGAT CTACCCCCAGTGGTGATAGAAA	61.5	78	103
*Nrf2*	NM_010902.4	Forward Reverse	CCCAGCAGGACATGGATTTGA AGCTCATAGTCCTTCTGTCGC	60	77.1	106
*Pd-l1*	NM_021893.3	Forward Reverse	CTCGCCTGCAGATAGTTCCC AGCCGTGATAGTAAACGCCC	60	77.4	94
*Sox9*	NM_011448.4	Forward Reverse	GTGAAGAACGGACAAGCGGA GATTGCCCAGAGTGCTCGC	60	84	148
*Vegf-α*	NM_001110268.1	Forward Reverse	GCAGATGTGAATGCAGACCAAA GCGTGGTGGTGACATGGTTA	59	83	154

## Data Availability

The original contributions presented in this study are included in the article/Supplementary Materials. Further inquiries can be directed to the corresponding author.

## Supplementary Materials

The following supporting information can be downloaded at: https://www.mdpi.com/article/10.3390/bioengineering13030336/s1, Figure S1: Schematic representation of the four in vitro models established in this study.; Figure S2: Invasion assay image segmentation using the Trainable Weka Segmentation plugin in Fiji.; Figure S3: Flow cytometry–based identification of macrophages in MCT spheroids. Figure S4: Validation of CRISPR/Cas9-mediated Nrf2 and Keap1 knockout in N-HCC25 cells. Figure S5: Characterization of the BMDMs used for MCT experiments. Figure S6: 3D-printed Transwell Tower construction. Table S1: qPCR protocol

## Abbreviations

The following abbreviations are used in this manuscript:

Arg1	Arginase 1
BMDMs	Bone marrow-derived macrophages
HCC	Hepatocellular carcinoma
KEAP1	Kelch-like ECH-associated protein 1
KO	Knock out
LCM	L929-conditioned medium
MCM	Macrophage conditioned media
MCTs	Multicellular tumor spheroids
N-HCC25	NASH-derived hepatocellular carcinoma 25 cell line
NASH	Non-alcoholic steatohepatitis
Nrf2	Nuclear factor erythroid 2-related factor2
Nqo1	NADPH dehydrogenase quinone 1
NTC	No-target control (sgRNA without mammalian target)
Pd-l1	Programmed death ligand 1
RT-qPCR	Reverse transcriptase-quantitative polymerase chain reaction
TAMs	Tumor-associated macrophages
TCM	TAM conditioned media
TIME	Tumor immune microenvironment
Vegf-α	Vascular endothelial growth factor A
WT	Wild type
