# Supplementary material for: Establishment of a 3D Multicellular HCC Tumor Spheroid Model to Unravel Nrf2’s Influence on the Tumor Immune Microenvironment
Source: Bioengineering (Basel). 2026 Mar 13;13(3):336. doi: 10.3390/bioengineering13030336 (PMC13023928; doi:10.3390/bioengineering13030336)
Supplement: Supplementary file 1 [file bioengineering-13-00336-s001.zip › Böttcher et al. 2026 - Establishment of a 3D multicellular HCC tumor spheroid_Appendix.pdf]

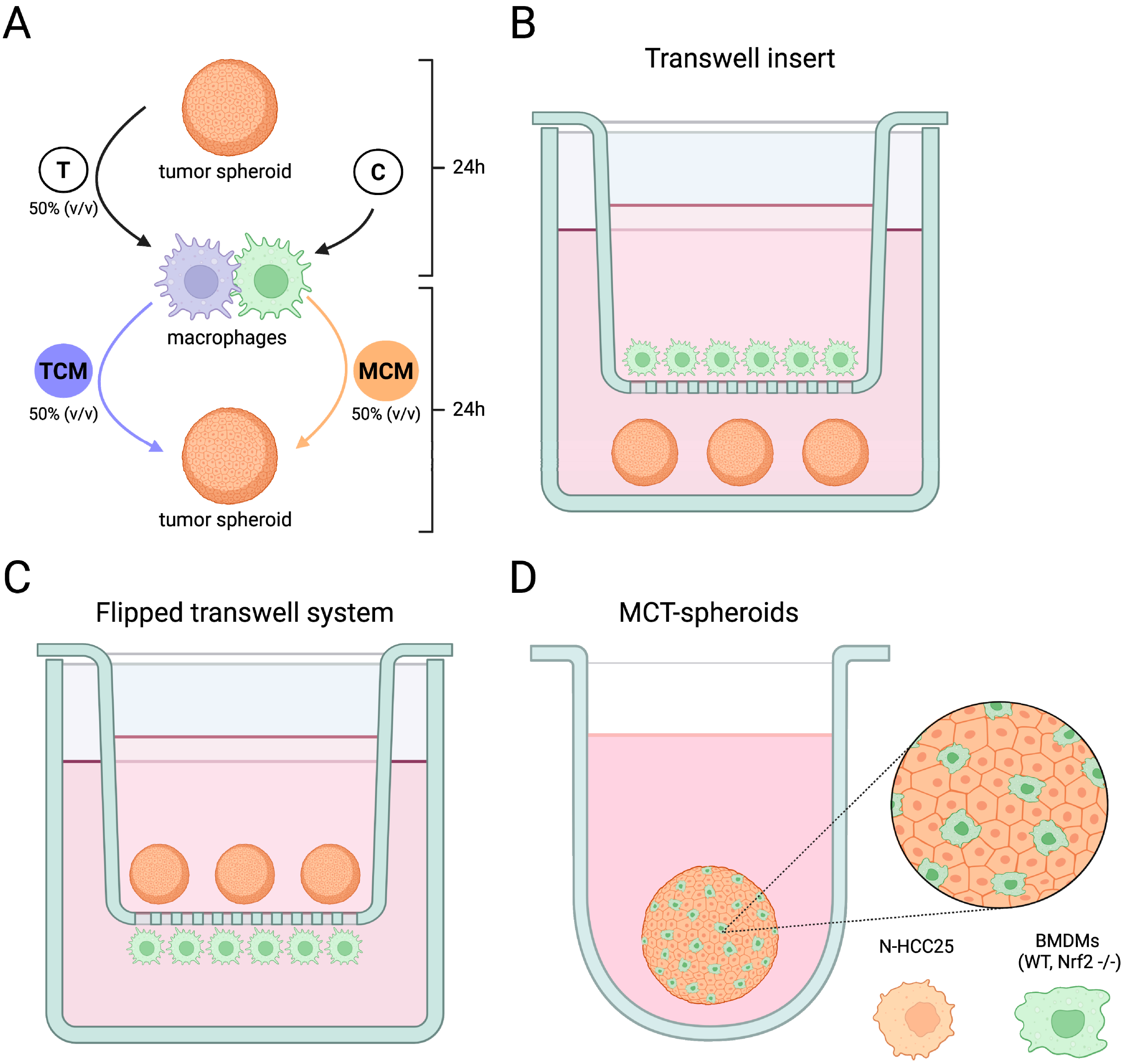

Figure S1. Schematic representation of the four *in vitro* models established in this study. (A) In the conditioned media approach, N-HCC25 tumor spheroids were incubated with supernatant derived from either naïve macrophages (MCM) or tumor-associated macrophages (TCM). (B) The transwell insert setup involved placing transwell inserts into 24-well plates where spheroids were cultured at the bottom of the well and macrophages were seeded inside the insert. (C) In the flipped transwell tower configuration, macrophages were seeded on the underside of the transwell membrane while N-HCC25 spheroids were placed inside the insert to allow closer proximity. (D) The direct co-cultivation model was established by mixing 7,500 N-HCC25 cells and 7,500 macrophages in U-shaped 96-well plates to form 3D multicellular tumor spheroids (MCT). Created in BioRender. Böttcher, N. (2026) https://BioRender.com/t44esyy

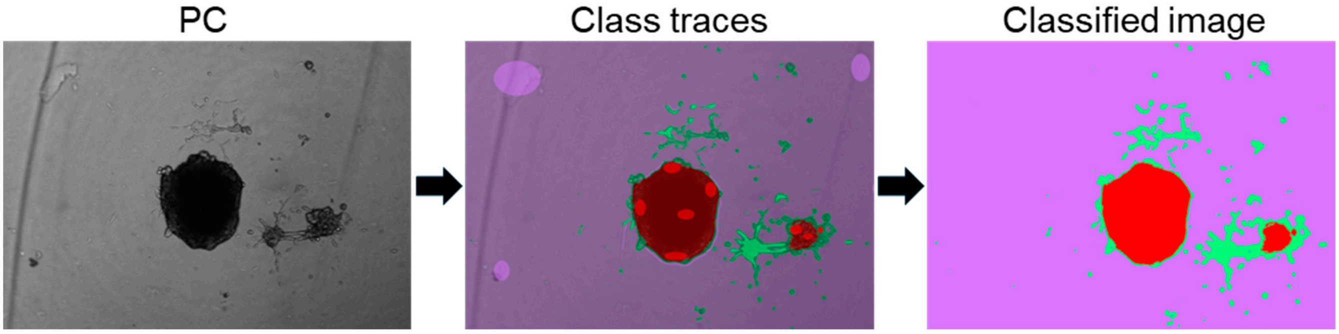

Figure S2. Invasion assay image segmentation using the Trainable Weka Segmentation plugin in Fiji. Left: phase-contrast image of spheroid invasion assay. Middle: representative manual annotations (traces) used for classifier training, indicating spheroid core (red), invasive protrusions (“spikes”, green), and background (purple). Right: resulting classified image showing the final segmentation output for the three classes.

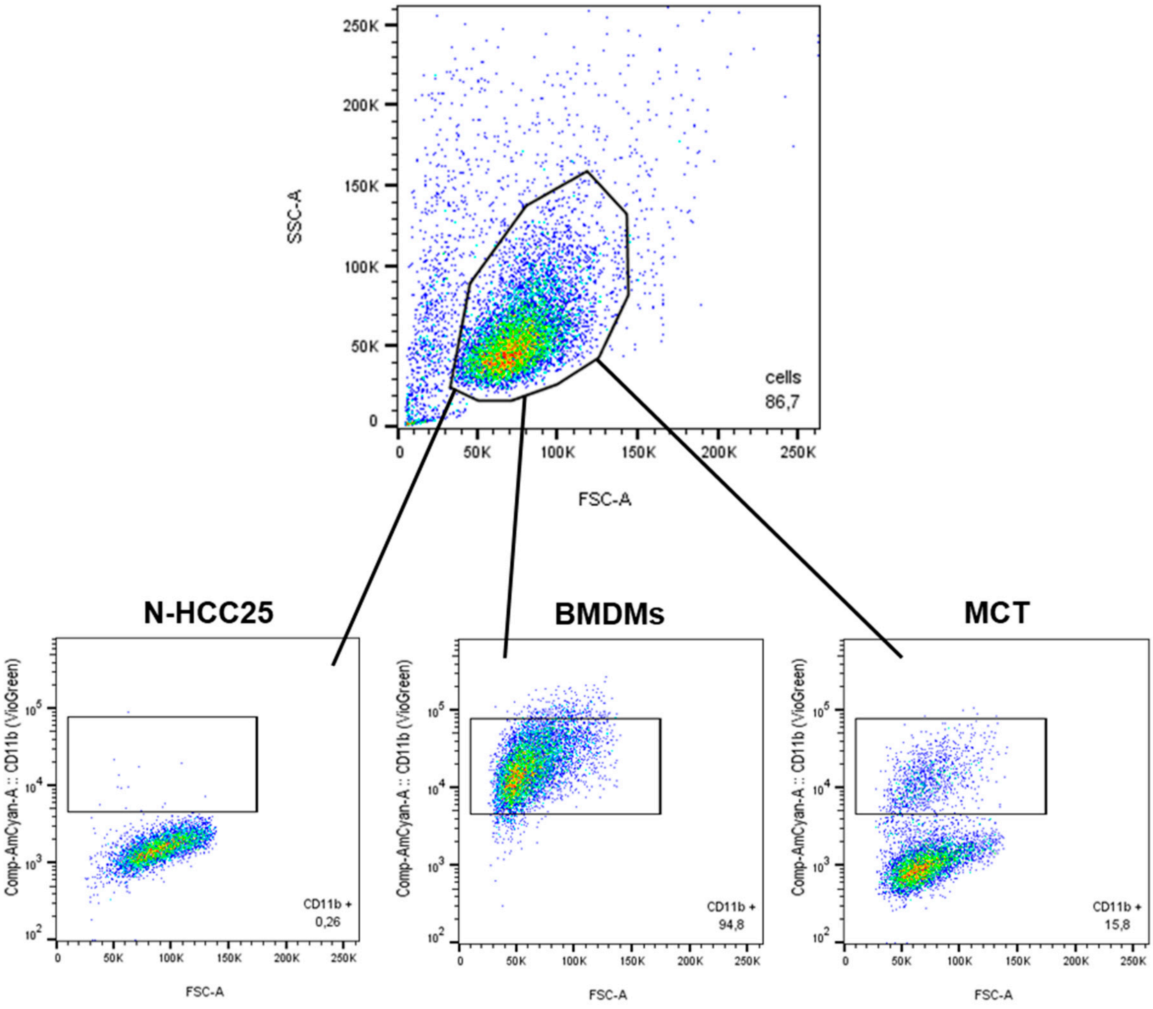

Figure S3. Flow cytometry–based identification of macrophages in MCT spheroids. Top: forward scatter area (FSC-A) versus side scatter area (SSC-A) used to exclude debris and define the main cell population. Bottom: gated cell populations from N-HCC25 monoculture spheroids, BMDMs, and MCT spheroids. Macrophages were identified based on CD11b^{+} expression. Percentages indicate the proportion of CD11b^{+} cells relative to the parent gate.

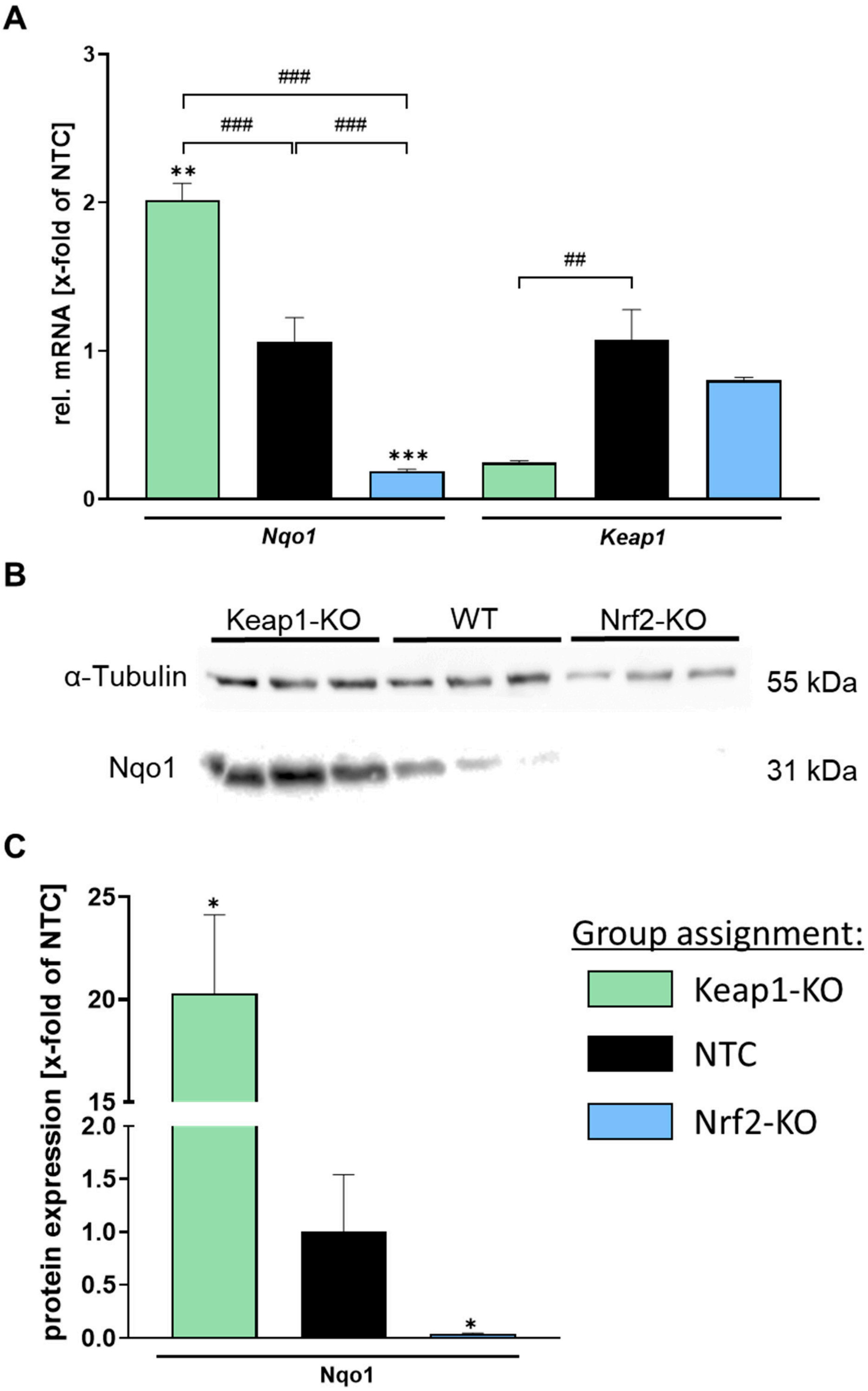

Figure S4. Validation of CRISPR/Cas9-mediated Nrf2 and Keap1 knockout in N-HCC25 cells. (A) Relative mRNA expression of Nqo1 and Keap1 were analyzed by RT-qPCR to verify the genetic modifications. Nqo1 expression served as a functional readout for Nrf2 activity. Gene expression was normalized to the reference genes Cul4a and Ywhaz. Data represent mean + SEM, n = 6. Statistical analysis was performed using a one-way ANOVA with Tukey's HSD post-hoc test. Statistical significance is indicated as * $p < 0.05$, ** $p < 0.01$, *** $p < 0.005$ compared to the WT control and # $p < 0.05$, ## $p < 0.01$, ### $p < 0.005$ for comparisons between the different genotypes. (B) Visualization of the validation on the protein level via western blot analysis of Nqo1 protein normalized to α-Tubulin. (C) Densitometry of the Nqo1 western blot experiment using Quantity One 4.6.9 software (BioRad Laboratories, Feldkirchen, Germay). Data represent mean + SEM, n = 3. Statistical analysis was performed using a one-way ANOVA with Tukey's HSD post-hoc test. Statistical significance is indicated as * $p < 0.05$ compared to the WT control.

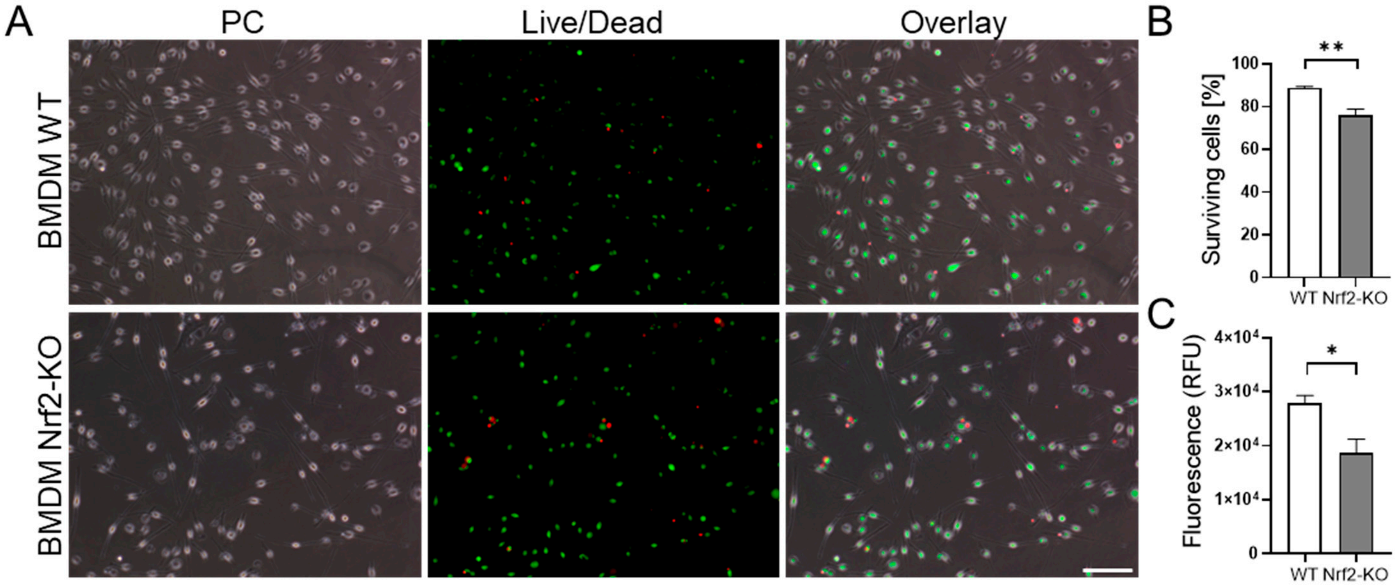

Figure S5. Characterization of the BMDMs used for MCT experiments. BMDMs were differentiated from bone marrow using L292-conditioned media for a period of 6 days. (A–B) For live dead staining $2{\times}10^5$ cells were seeded into a 6-well plate. Live dead assay was performed as described by the manufacturer and 3 positions in the well were selected randomly and counted utilizing the Fiji cell counter plugin. Data represents mean + SEM, n = 3. (C) For the CellTiter Blue assay, $2{\times}10^4$ BMDMs were seeded into a 96-well plate. The assay was performed as described by the manufacturer. Data represents mean + SEM, n = 8. Statistical analysis was performed using a student's t-test. Statistical significance is indicated as * $p < 0.05$, ** $p < 0.01$ compared to the WT control.

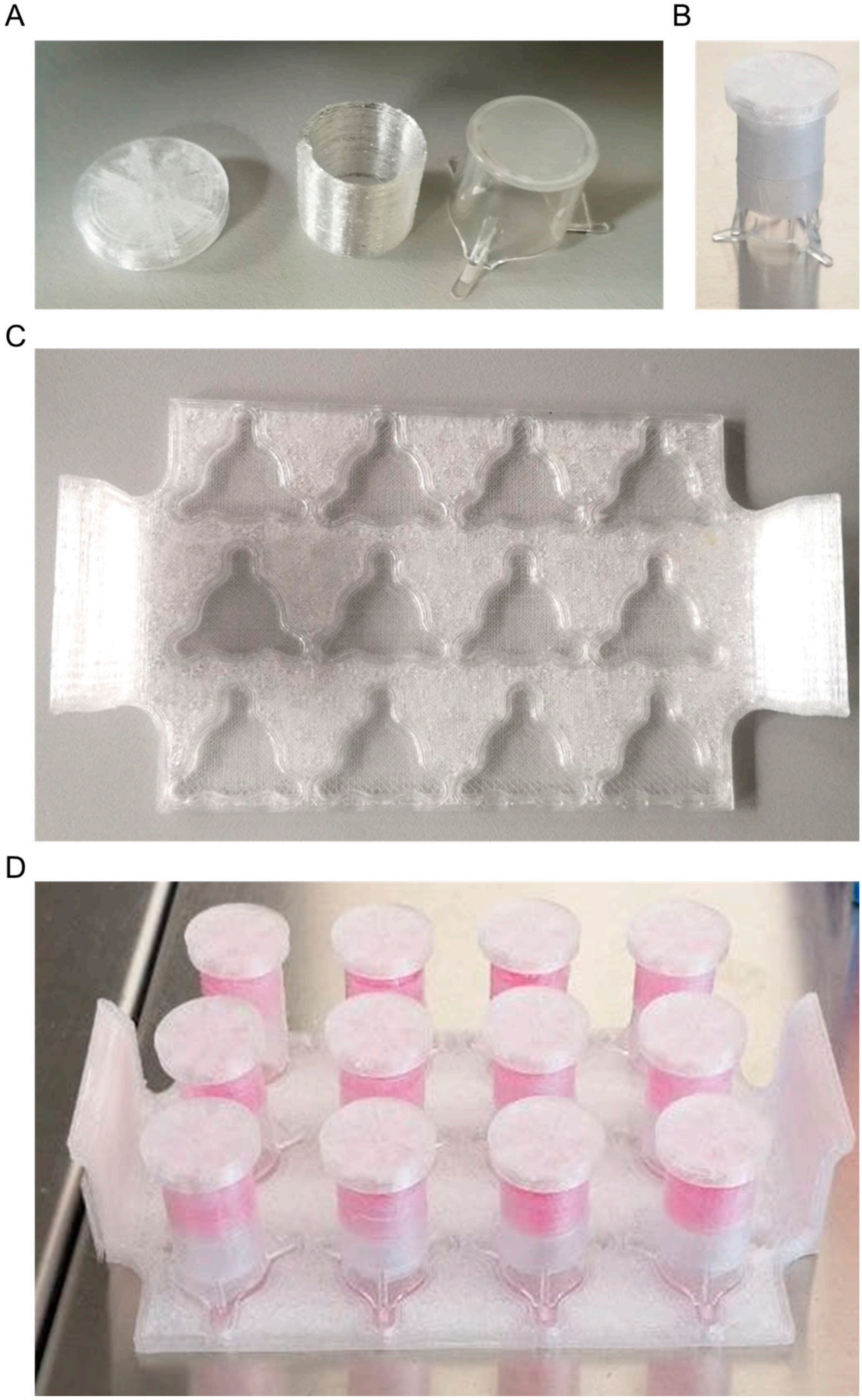

Figure S6. 3D-printed Transwell Tower construction. (A) Depiction of the ThinCert® transwell insert, the 3D-printed ring and the lid. (B) Assembled Transwell Tower with the ring attached to the ThinCert® insert and the lid on top. (C) 3D-printed tray with precise cavities for inserting a total of 12 Transwell Towers for easier handling and transport. (D) Representative image of a full tray with 12 Transwell Towers incubating BMDMs on the bottom side of the ThinCert® membrane.

Table S1. qPCR protocol

**Step**	**Temperature [°C]**	**Time [min:sec]**
HotStart Activation	50.0	02:00
Initial ds separation	95.0	10:00
Cycles (40x)	95.0	00:15
	s. Table 2 (T_A)	00:30
	72.0	00:30
Melt curve	60.0	01:00
	Increment 0.1 (up to 95.0)	
